# Verbal learning and hippocampal dysfunction in schizophrenia: A *meta*-analysis

**DOI:** 10.1016/j.neubiorev.2017.12.001

**Published:** 2018-03

**Authors:** Mathilde Antoniades, Tabea Schoeler, Joaquim Radua, Isabel Valli, Paul Allen, Matthew J. Kempton, Philip McGuire

**Affiliations:** a Department of Psychosis Studies, King’s College London, Institute of Psychiatry, Psychology and Neuroscience, London, UK; b FIDMAG Germanes Hospitalàries – CIBERSAM, Sant Boi de Llobregat, Barcelona, Spain; c Centre for Psychiatric Research and Education, Department of Clinical Neuroscience, Karolinska Institutet, Stockholm, Sweden; d Department of Psychology, University of Roehampton, London, UK

**Keywords:** Cognition, Volume, MRI, Memory, Schizophrenia, Medial temporal lobe

## Abstract

This *meta*-analysis summarizes research examining whether deficits in verbal learning are related to bilateral hippocampal volume reductions in patients with or at risk for schizophrenia and in healthy controls. 17 studies with 755 patients with schizophrenia (SCZ), 232 Genetic High Risk (GHR) subjects and 914 healthy controls (HC) were included. Pooled correlation coefficients were calculated between hemisphere (left, right or total) and type of recall (immediate or delayed) for each diagnostic group individually (SCZ, GHR and HC). In SCZ, left and right hippocampal volume positively correlated with immediate (r = 0.256, 0.230) and delayed (r = 0.132, 0.231) verbal recall. There was also a correlation between total hippocampal volume and delayed recall (r = 0.233). None of these correlations were significant in healthy controls. There was however, a positive correlation between left hippocampal volume and immediate recall in the GHR group (r = 0.356). The results suggest that hippocampal volume affects immediate and delayed verbal learning capacity in schizophrenia and provides further evidence of hippocampal dysfunction in the pathophysiology of schizophrenia.

## Highlights

• Verbal learning is one of the most impaired cognitive functions in schizophrenia.
• Novel method for including unreported non-significant effects in the *meta*-analysis.
• Positive correlations between verbal learning and hippocampal volume in SCZ.
• Absent correlations in healthy controls.

## 1 Introduction

Schizophrenia (SCZ) is a complex disorder with widespread neuroanatomical and neurofunctional deficits. It is associated with a substantial cognitive impairment that affects working memory, processing speed, attention and verbal memory (Heinrichs and Zakzanis, 1998, Dickinson et al., 2007, Fioravanti et al., 2005, Mesholam-Gately et al., 2009). Such cognitive deficits have been shown to be present in children and adolescents who later develop schizophrenia, long before they develop any overt symptoms (Reichenberg et al., 2010). Cognitive deficits have also been detected in the prodromal stage of the illness (Bora and Murray, 2014) and in first-episode patients (Mesholam-Gately et al., 2009). Furthermore, studies have consistently found that verbal learning has shown the greatest impairment throughout the disease stages (Massuda et al., 2013, Mesholam-Gately et al., 2009, Stone et al., 2011, Lencz et al., 2006, Silver and Bilker, 2015).

The most common way to assess verbal learning is through the use of word list learning tasks such as the Rey Auditory Verbal Learning Task (RAVLT, Lezak et al., 2004), the California Verbal Learning task (Delis et al., 1987), the Hopkins Verbal Learning Test (Brandt and Benedict, 2001) or with the Logical Memory Stories test of the Wechsler Memory Scale (Wechsler, 1987). All of these tasks provide a measure of immediate and delayed recall. Factor analysis of the RAVLT revealed three basic factors: acquisition, storage and retrieval (Vakil and Blachstein, 1993). Thus, measures of immediate recall may reflect the cellular process of encoding and delayed recall may reflect the processes of storage and retrieval (Delis et al., 1991).

Verbal learning and memory relies on a network of regions including the prefrontal cortex and medial temporal lobe (MTL) structures, where the hippocampus is important for the acquisition of new information (Simons and Spiers, 2003, Preston and Eichenbaum, 2013). More specifically, studies have shown that the CA2, CA3 and dentate gyrus were activated during encoding of face-name and object-name pairs whereas the subiculum was activated during retrieval (Zeineh et al., 2003, Eldridge et al., 2005). In shorter time periods, the hippocampus is thought to aid retrieval by reactivating neurons that were involved in learning (Tanaka et al., 2014). The combination of these findings and results from many other studies (Kircher et al., 2008, Davachi and Wagner, 2002, Duncan et al., 2014, Hunsaker and Kesner, 2013, Kesner, 2007) highlight the importance of the hippocampus in the encoding, storage and retrieval of memories.

The hippocampus is thought to be one of the key regions implicated in the pathophysiology of schizophrenia (Lodge and Grace, 2011). Studies consistently report hippocampal volume reductions in patients with schizophrenia in comparison to healthy controls (HC, Van Erp et al., 2016, Adriano et al., 2012, Steen et al., 2006); a finding that is also reported in high risk individuals (Wood et al., 2010, Witthaus, 2010; Phillips et al., 2002). Furthermore, an examination into the functioning of the regions involved in verbal learning has shown that patients with schizophrenia (Jessen et al., 2003, Weiss et al., 2004) and those at risk for psychosis (Allen et al., 2011, Allen et al., 2012) have reduced activation in both prefrontal and MTL structures. Thus, abnormalities in hippocampal integrity and function could be related to some of the cognitive deficits observed in patients with or at risk for schizophrenia.

Based on the presence of both memory deficits and hippocampal abnormalities in patients with schizophrenia, many researchers examined the link between verbal learning performance and hippocampal volume. These studies report positive (Herold et al., 2015, Sanfilipo et al., 2002) and negative (Thoma et al., 2009) correlations but the majority report non-significant findings. The aim of this *meta*-analysis is to pool the results from these studies, which often have small sample sizes, in order to reveal the relationship between verbal learning and hippocampal volume in patients with or at risk for schizophrenia.

Our hypotheses were threefold. First, based on the findings showing that patients with schizophrenia have impairments in verbal learning and that they have hippocampal volume reductions, we hypothesised that there would be a positive correlation between bilateral hippocampal volume and verbal learning performance in patients with schizophrenia. Second, based on the *meta*-analytic findings that verbal encoding more so than retrieval is lateralised to the left hippocampus (Persson and Soderlund, 2015), we hypothesised that correlations would be between immediate recall (as a measure of encoding) and left hippocampal volume. Third, we hypothesised that positive correlations would also exist in subjects at risk for schizophrenia, mirroring those in the schizophrenia group.

## 2 Methods

### 2.1 Search strategy

**Fig. 1:**
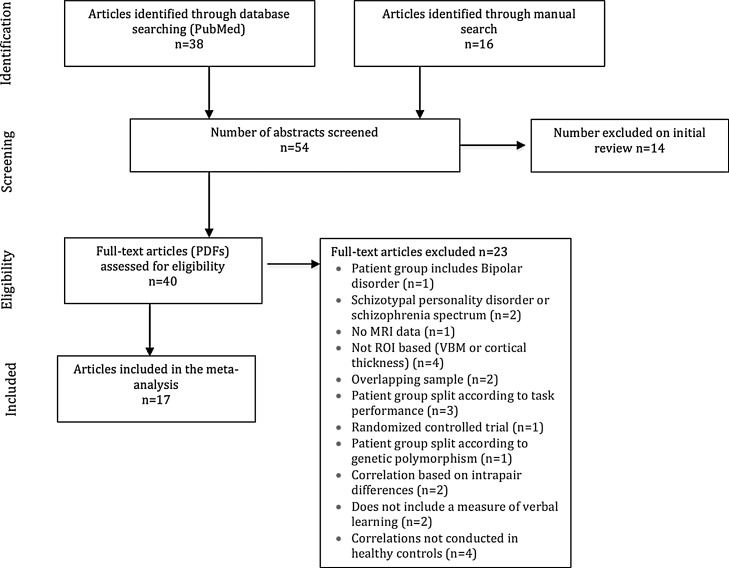
Flow chart of the screening and selection process.

A systematic literature search strategy was conducted between January 1st 1980 and June 16th 2016 following the Preferred Reporting Items for Systematic Reviews and Meta-Analyses (PRISMA) guidelines (Moher et al., 2009). The PubMed database was used to identify relevant publications with the following search terms: “schizophrenia”, “psychosis”, “hippocamp*”, “medial temporal lobe”, “volume”, “verbal”, “neuropsych*”, “learning” and “recall”. All returned articles were screened and all texts other than original articles were excluded (e.g. reviews). The remaining studies were then assessed for eligibility and finally for inclusion criteria. We also examined the bibliographies of the included articles and the main reviews on this topic to identify any additional studies for inclusion. A flow chart of the selection process for this *meta*-analysis is shown in Fig. 1.

### 2.2 Selection criteria

Studies were eligible for inclusion if they: 1) were original articles written in English; 2) compared patients with DSM-III (APA, 1980) or later diagnosis of schizophrenia spectrum disorders or clinical (Fusar-Poli et al., 2013) or genetic (Johnstone et al., 2000) high risk state for psychosis with healthy controls; 3) used a region of interest approach with either manual or automatic segmentation of the hippocampus (studies using whole-brain voxel based morphometry methods were excluded); 4) directly correlated hippocampal volume and measures of verbal learning and 5) correlations were assessed in both patient and control groups. Studies were excluded if: 1) the patient group included diagnoses other than schizophrenia (e.g. Bipolar disorder) or 2) there were overlapping samples (i.e. data from the same participants were used in two or more separate studies), in which case the study with the smaller data set was excluded (Herold et al., 2013, Nestor et al., 2007).

### 2.3 Recorded variables

Simple and partial correlation coefficients between the verbal learning task and hippocampal volume were extracted by one author (M.A) and independently verified by another (T.S). The following variables were also recorded from each article: reference, publication year, sample size, age, percentage of males, task, illness stage, percentage of SCZ sample taking antipsychotic treatment, hippocampal region, method of segmentation, field strength, type of correlation and correlation covariates. Wherever there was missing data, the authors were contacted (10 were contacted and 3 provided missing information).

### 2.4 Statistical analysis

After contact with the authors, we did not know the specific correlation coefficients of a number of studies but we knew that they had not reached statistical significance. In other words, these correlations were non-statistically significant unreported effects (NSUEs), (SCZ: Haukvik et al., 2015 and HC: Lappin et al., 2014, Guo et al., 2014, Rametti et al., 2007, Karnik-Henry et al., 2012, Kuroki et al., 2006, Sanfilipo et al., 2002). Importantly, studies with NSUEs cannot be excluded as this would bias the *meta*-analysis towards the probably larger correlations reported in the non-NSUEs studies; note that studies finding larger, statistically-significant effects usually report these effects, whereas studies finding smaller, non-statistically significant effects may not report them. In order to overcome this bias and correctly include studies with NSUEs, we used a novel method called MetaNSUE developed and validated in Radua et al., 2015. Briefly, this method calculates upper and lower bounds where the Fisher z-transformations of the unreported correlations should be according to the alpha levels used in the studies. Then the Fisher z-transformations of the NSUEs are multiply imputed within these thresholds according to the Fisher z-transformation of the most likely correlation coefficient and the most likely between-study heterogeneity. In each set of imputations, a restricted-maximum likelihood random-effects model is used to calculate a *meta*-analytic Fisher z-transformation, and the Fisher z-transformation of the different imputation sets are finally pooled to obtain a single correlation coefficient (more detail can be found in Radua et al., 2015). Note that random-effects models account for the heterogeneity between studies, e.g. due to the use of different tasks and different segmentation methods; the percentage heterogeneity that is not due to sampling error was measured with the I^2^ statistic. Potential reporting bias was assessed by *meta*-regressing the Fisher z-transformations of the correlation coefficients by their standard error to identify funnel plot asymmetry and thus show if small studies may have been published only if they had significant results. This test was only done in *meta*-analyses with more than 10 studies because otherwise the test does not have sufficient power to differentiate between chance and genuine asymmetry (Sterne et al., 2011). Finally, leave-one-out jackknife sensitivity analyses were conducted to see whether a single study might be driving the results.

Studies that provided correlations in sub-regions of the hippocampus (e.g. anterior and posterior hippocampus) were considered as repeated measures and the weights attributed to them were adjusted accordingly with MetaNSUE. This technique avoids the subjective choice of one sub-region over another which could introduce bias into the *meta*-analysis. Gur et al., 2000 provided separate correlations for men and women, these were included as independent datasets and their weights were adjusted. These adjustments depend on the expected correlation between the repeated measures, which by default is assumed to be 0.3; thus in order to check whether the *meta*-analysis results could be influenced by this assumption, we ran a sensitivity analysis with the correlation coefficient set to 0 and 1.

Given that simple and partial correlations (the latter is a correlation between two variables whilst adjusting for a third variable) each have their own advantages and disadvantages in terms of comparability or control of potential confounds, we conducted the *meta*-analysis three times: a) using a combination of simple and partial correlations, depending on the type reported by the study; b) using simple correlations and discarding those studies from which only partial correlations could be obtained; and c) using partial correlations only.

Similarly, separate *meta*-analyses were run for left, right and total hippocampal volume and for immediate and delayed recall in SCZ and HC. Details of the studies included in each *meta*-analysis can be found in Table 1 (methodology details of these studies can be found in Table 1 of the Supplementary Material). For the single study reporting findings using the CVLT (Haukvik et al., 2015), the correlation between hippocampal volume and free recall was used (as opposed to the results with cued recall) because this is more comparable to the method of recall in the other verbal learning tasks. Studies using composite scores (a combination of several different verbal learning tasks) were included in the “delayed” category (Karnik-Henry et al., 2012, Sanfilipo et al., 2002, Sachdev et al., 2000).

**Table 1:** Description of the demographic, cognitive and neuroimaging measures and covariates of the samples included in the *meta*-analysis.

Study	Task	Region	Covariates	Stage	n	Age (mean)	Males (%)	AP Med (%)	n	Age (mean)	Males (%)
Immediate recall					Schizophrenia or GHR				Healthy Controls		
Herold et al. (2015)	WMS-R LM I	L/R Hipp, Ant & Post	Edu	SCZ	25 old	55.92	76	88	21	53.67	57.1
					23 young	32.78	60.9	100			
Guo et al. (2014)	HVLT total immediate	L/R Hipp	Age, sex, Edu, PANSS total	FEP	51	22.5	64.7	0	41	22.8	58.5
Francis et al. (2013)	WMS-R LM I	L/R Sub	None	GHR	46	25	30.4	0	30	24	43.3
Killgore et al. (2009)	WMS-R LM I	L/R Hipp	None	SCZ	19	34.3	73.68	100*	20	27.4	90
Thoma et al. (2009)	WMS-R LM I	L/R Ant & Post	ICV	SCZ	24	40.09	83.33	100	24	41.74	66.67
Exner et al., (2008)	WMS-R LM I	L/R Hipp	None	SCZ	21	31	66.67	100	21	29	61.9
Seidman et al. (2002)	WMS-R LM I	L/R Hipp	Sex, group, sex*group, Hand, Eth, parental Edu, IQ	GHR	28 simplex	41.9	35.7	0	48	40.1	56.3
					17 multiplex	38.9	41.2				

Delayed recall					Schizophrenia or GHR				Healthy Controls		
Herold et al. (2015)	WMS-R LM II	L/R Hipp, Ant & Post	Edu	SCZ	25 old	55.92	76	88	21	53.67	57.1
					23 young	32.78	60.9	100			
Haukvik et al. (2015)	CVLT delayed free recall	Total Sub	None	SCZ	177	31.6	61	84.7	261	36	52
Lappin et al. (2014)	RAVLT trial 7	L/R Hipp	None	FEP	90				83		
Francis et al. (2013)	HVLT total	L/R Sub	None	GHR	46	25	30.4	0	30	24	43.3
Karnik-Henry et al. (2012)	WMS-R LM I, LM II & CVLT composite	L Hipp	Sex	SCZ	36	22.5	84.6	84.6	46	21.1	56.52
				GHR	31	22.1	45.5	0	49	20.4	28
Killgore et al.(2009)	WMS-R LM II	L/R Hipp	None	SCZ	19	34.3	73.68	100*	20	27.4	90
Thoma et al. (2009)	WMS-R LM II	L/R Ant & Post	ICV	SCZ	24	40.09	83.33	100	24	41.74	66.67
Exner et al., 2008	WMS-R LM II	L/R and total Hipp	None	SCZ	21	31	66.67	100	21	29	61.9
Rametti et al. (2007)	RAVLT delayed	L Ant	None	SCZ	28	27.7	75	100	33	28.09	63.64
Kuroki et al. (2006)	WMS-R LM II	L/R Hipp	MD	SCZ	24	40.3	100	100	31	40.6	100
Toulopoulou et al. (2004)	WMS-R LM II	L Hipp	Age, gender, group	SCZ	56	32.8	69.7	100	55	38.8	49.1
				GHR	90	50.2	37.8	0			
Seidman et al. (2002)	WMS-R LM II	L/R Hipp	Sex, group, sex*group, Hand, Eth, parental Edu, IQ	GHR	28 simplex	41.9	35.7	0	48	40.1	56.3
					17 multiplex	38.9	41.2				
Sanfilipo et al. (2002)	WAIS, WMS-R LM I, LM II, Buschke	L/R Hipp	ICV, age	SCZ	62	38.8	100	100	27	35.7	100
O’driscoll et al. (2001)	WMS-R LM II	L/R and total Am/Hipp	None	GHR	20	36.2	45	0	14	35.4	36
Gur et al. (2000)	WMS-R LM I, LM II & CVLT (1–5)	Total Hipp	None	SCZ	100	29.2	58	61	110	26.1	51
Sachdev et al. (2000)	LM1 &2, VR, WAIS, COWAT, WCST	L/R Hipp	None	SCZ	20	64.4	60	100	24	72.67	79

WMS-R: Wechsler memory scale-revised, LM I: logical memory 1 (i.e. immediate recall), LM II: logical memory 2 (i.e. delayed recall), SCZ: schizophrenia; AP Med: antipsychotic medication, GHR: genetic high risk; RAVLT: Rey Auditory Verbal Learning Task, HVLT: Hopkins verbal learning task, ICV: intracranial volume, L/R: left/right, Sub: subiculum, Hipp: hippocampus, Am: amygdala, Ant: anterior, Post: posterior, Edu: education, Eth: ethnicity, MD: medication dosage, Hand: handedness, * denotes that all patients were medicated but the study does not specify the type of medication.

Although we set out to study the structural-behavioural relationship in individuals at clinical and familial risk of schizophrenia, the search did not reveal any studies examining these correlations in subjects at clinical high risk for schizophrenia. Therefore the *meta*-analysis was conducted on the five studies that reported correlations in participants with a genetic risk for psychosis (Francis et al., 2013, Toulopoulou et al., 2004, Karnik-Henry et al., 2012).

In order to examine the effect of antipsychotic medication on the correlation coefficients, a *meta*-regression was conducted using the proportion of each study’s SCZ sample taking antipsychotic medication. A *meta*-regression was also conducted with age in both SCZ and HC, however there was insufficient data to examine the effect of the number of years spent in education.

**Fig. 2:**
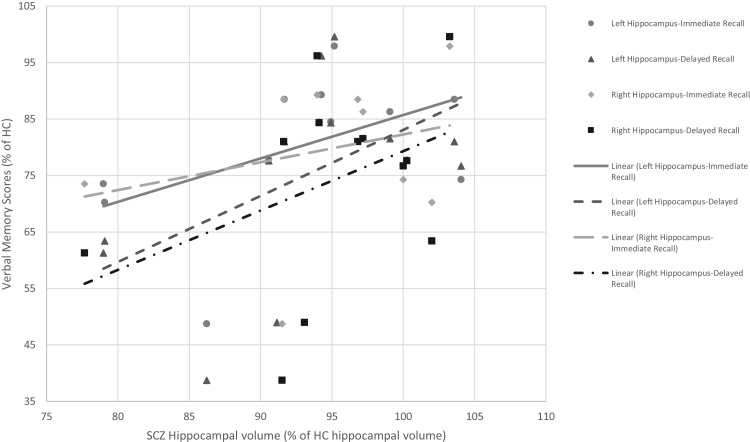
Graph showing that verbal learning in patients with schizophrenia and their relatives is 55–90% that of healthy controls whereas hippocampal volume is 75–105% that of controls. Memory scores and hippocampal volumes of healthy controls were all set to 100% and are not shown on the graph.

Wherever possible, mean left and right hippocampal volume data was extracted for HC, SCZ and genetic high risk (GHR) groups and plotted against mean immediate or delayed memory score (raw scores were used except for Francis et al., 2013) to examine how the relationship between these variables in SCZ and GHR groups compared to that in HC (this data is shown in Fig. 2). The data used to compile the graphs is shown in Table 2 of the Supplementary Material but has been converted so that SCZ and GHR values are a percentage of the HC values. Data was only available for 8 studies.

**Table 2:** Comparison of MetaNSUE findings using combined, simple and partial correlation coefficients in patients with schizophrenia and healthy controls.

Analysis (group, hemisphere, type of recall)	Simple and partial correlations combined					Simple correlations only					Partial correlations only				
	Number of independent studies (number of correlations)	Number of NSUEs	Total* number of subjects	Meta-analysis result: Correlation coefficient (*p* value)	95% CI	Number of independent studies (number of correlations)	Number of NSUEs	Total* number of subjects	Meta-analysis result: Correlation coefficient (*p* value)	95% CI	Number of independent studies (number of correlations)	Number of NSUEs	Total* number of subjects	Meta-analysis result: Correlation coefficient (*p* value)	95% CI
SCZ left immediate	5 (8)	0	162	0.256 (0.0029)	0.089 to 0.409	3 (4)	0	63	0.146 (0.24)	-0.095 to 0.371	3 (6)	0	123	0.242 (0.097)	−0.045 to 0.493
SCZ left delayed	11 (15)	0	431	0.131 (0.0038)	0.042 to 0.218	7 (8)	0	229	0.179 (0.0304)	0.017 to 0.332	6 (10)	0	250	0.153 (0.0464)	0.002 to 0.297
SCZ right immediate	5 (8)	0	162	0.230 (0.001)	0.094 to 0.358	3 (4)	0	63	0.174 (0.15)	−0.066 to 0.396	3 (6)	0	123	0.128 (0.37)	−0.150 to 0.387
SCZ right delayed	8 (12)	0	311	0.234 (<0.0001)	0.135 to 0.329	6 (7)	0	201	0.256 (0.0013)	0.102 to 0.398	4 (8)	0	158	0.227 (0.093)	−0.038 to 0.462
HC left immediate	7 (10)	1	204	0.019 (0.87)	−0.207 to 0.243	4 (5)	0	95	−0.122 (0.43)	−0.402 to 0.178	4 (7)	1	133	0.248 (0.0073)	0.068 to 0.412
HC left delayed	15 (19)	7	519	0.065 (0.26)	−0.049 to 0.177	8 (9)	2	243	0.103 (0.24)	−0.068 to 0.267	8 (12)	5	302	0.075 (0.32)	−0.072 to 0.219
HC right immediate	7 (10)	1	204	0.063 (0.57)	−0.152 to 0.272	4 (5)	0	95	−0.081 (0.62)	−0.379 to 0.233	4 (7)	1	133	0.297 (0.0007)	0.128 to 0.449
HC right delayed	11 (15)	4	336	0.123 (0.0699)	−0.01 to 0.251	7 (8)	1	210	0.095 (0.32)	−0.093 to 0.277	5 (9)	3	150	0.207 (0.0235)	0.028 to 0.372

*Total only includes the number of subjects from repeated measures once, NSUE: non-significant unreported effect, SCZ: schizophrenia, HC: healthy control, CI: confidence interval.

All analyses were performed in R version 3.3 with the MetaNSUE package (http://www.metansue.com/).

In order to test the hypothesis that the correlation between immediate recall and left hippocampal volume was stronger than with the right hippocampal volume, correlation coefficients were transformed into z scores (“Fisher’s r to z transformation”) and the z test statistic was used to determine statistical significance.

## 3 Results

The details of the analyses along with all the results for patients with schizophrenia and healthy controls are shown in Table 2. Forest plots for the main analyses are presented in the Supplementary Material (Figs. 1–8).

### 3.1 Meta-analyses in patients with schizophrenia

The analyses run using simple and partial correlations (Forest plots for these analyses are in Figs. 1–4 in the Supplementary material) revealed significant positive correlations between left and right hippocampal volume and both immediate and delayed recall (I^2^ = 26.93%, 0%, 0.8% and 1.04%, respectively). Jackknife analyses were run for all analyses and showed similar correlations when any single study was discarded. Potential reporting bias was only examined for the Left Hippocampus-Delayed Recall analysis but none was observed (*P* = 0.099). Only the correlation between left and right hippocampal volume and delayed recall were significant in the *meta*-analysis including simple correlations whereas the *meta*-analysis including partial correlations only revealed a significant correlation between left hippocampal volume and delayed recall.

The correlation between left hippocampal volume and immediate recall was not significantly greater than the correlation between right hippocampal volume and immediate recall (*Z*_test_ = 0.25, *P* = 0.401).

A *meta*-analysis was conducted on studies that provided correlations between total hippocampal volume and delayed recall (other correlations were not available). This analysis included 4 correlations from 2 studies with reported correlations (Exner et al., 2008, Gur et al., 2000: separate correlations were available for males and females) and 1 study (Haukvik et al., 2015) with an NSUE (the total sample size was n = 293). There was a significant positive correlation between delayed verbal recall and total hippocampal volume in patients with schizophrenia (r = 0.228; *P* = 0.0138; 95% CI, 0.047-0.394; I^2^ = 14.35%). Jackknife analyses revealed a loss of significance when a correlation by (Gur et al., 2000) was discarded (r = 0.163, *P* = 0.0516). Individual removal of all other studies did not affect the correlations (r range, 0.244–0.297 and *P* range, 0.005–0.035).

### 3.2 Meta-analyses in healthy controls

The analyses between left and right hippocampal volume and immediate and delayed recall were all non-significant in healthy controls (I^2^ = 57.09%, 7.58%, 54.51% and 9.63%, respectively). Jackknife analyses showed similar correlations when any single study was discarded in all analyses except for the Right Hippocampus-Delayed recall. There, they showed a significant correlation when Killgore et al. (2009) was discarded (r = 0.159, *P* = 0.02), when (Exner et al., 2008) was discarded (r = 0.150, *P* = 0.03) and when a correlation from (Herold et al., 2015) was discarded (r = 0.129, *P* = 0.048). No potential reporting bias was observed for the Left or the Right Hippocampus-Delayed Recall analyses (*P* = 0.54, 0.87; respectively). Forest plots of the analyses using combined simple and partial correlations are shown in Figs. 5 –8 in the Supplementary material. The correlations remained non-significant when only simple correlations were *meta*-analysed but became significant when only considering the partial correlations.

The *meta*-analysis between total hippocampal volume and delayed recall included 5 correlations from 4 studies with reported correlations (Haukvik et al., 2015, Exner et al., 2008, O’driscoll et al., 2001, Gur et al., 2000) and one study with an NSUE (Gur et al., 2000), (total sample size, n = 387). There was no correlation between total hippocampal volume and delayed verbal recall in healthy controls (r = 0.104; *P >* 0.05; 95% CI, −0.065-0.267; I^2^ = 15.01%). Jackknife analyses showed similar correlations when each study was discarded (r range, 0.016–0.149 and *P >* 0.05 in all cases).

### 3.3 Meta-analysis in GHR

The *meta*-analysis between left hippocampal volume and immediate recall (Francis et al., 2013, Seidman et al., 2002) revealed a significant positive correlation in GHR subjects (n = 89, r = 0.356; *P* = 0.0009; 95% CI, 0.153-0.531; I^2^ = 0%), however this analysis has a very small number of studies. Jackknife analyses showed similar correlations when each study was discarded (r range, 0.331–0.398 and *P* < 0.05 in all cases).

There were no significant correlations between left hippocampal volume and delayed recall (n = 228, r = −0.031; *P* > 0.05; 95% CI, −0.223-0.163; I^2^ = 32.17%) or between right hippocampal volume and immediate (n = 89, r = 0.160; *P >* 0.05; 95% CI, −0.075-0.379; I^2^ = 10.95%) or delayed recall (n = 107, r = −0.034; *P >* 0.05; 95% CI, −0.237-0.171; I^2^ = 4.36%) in GHR subjects. Jackknife analyses showed similar correlations when each study was discarded in each analysis.

The number of studies examining the correlation between total hippocampal volume and measures of recall in GHR subjects was too small to permit a *meta*-analysis.

### 3.4 Sensitivity analysis

No changes were observed on any *meta*-analysis result when the value of the parameter was set to 0 or 1.

### 3.5 Meta-regression

The *meta*-regression revealed a significant but very weak negative correlation between correlation coefficients for the Left Hippocampus-Immediate Recall analysis and the proportion of the SCZ sample taking antipsychotic medication (*r* = −0.004, *P* = 0.03, n = 5 studies). Data for the proportion of the SCZ sample on antipsychotic medication was unavailable for one study (Lappin et al., 2014) however no significant correlations were found for all other *meta*-analyses (Left Hippocampus-Delayed Recall: *r* = −0.009, *P >* 0.05, n = 10 studies; Right Hippocampus-Immediate Recall: *r* = 0.001, *P >* 0.05, n = 5 studies; Right Hippocampus-Delayed Recall: *r* = −0.016, *P >* 0.05, n = 7 studies).

Age was not related to correlation coefficients of any of the *meta*-analyses in patients with schizophrenia (Left Hippocampus-Immediate Recall: *r* = −0.014, *P* = 0.07, n = 5 studies; Left Hippocampus-Delayed Recall: *r* = 0.003, *P* = 0.619, n = 10 studies; Right Hippocampus-Immediate Recall: *r* = 0.008, *P* = 0.286, n = 5 studies; Right Hippocampus-Delayed Recall: *r* = 0.012, *P* = 0.126, n = 7 studies) but was significantly correlated with correlation coefficients for Right Hippocampus-Immediate Recall analysis in healthy controls (*r* = 0.014, *P* = 0.049, n = 7 studies). All other regressions in healthy controls were non-significant (Left Hippocampus-Immediate Recall: *r* = 0.014, *P* = 0.09, n = 7 studies; Left Hippocampus-Delayed Recall: *r* = 0.004, *P* = 0.436, n = 13 studies; Right Hippocampus-Delayed Recall: *r* = 0.005, *P* = 0.401, n = 10 studies).

## 4 Discussion

Here we present findings that show a significant positive correlation between bilateral hippocampal volume and the ability to recall verbal information immediately and after a short delay in patients with schizophrenia. Thus, as hippocampal volume decreases, verbal recall performance worsens. Furthermore, contrary to the hypothesis, although the correlation between the left hippocampus and immediate recall is qualitatively larger than the right, there was no statistically significant difference between the two.

There were no correlations between hippocampal volume and verbal learning in healthy controls. However, some correlations in the healthy control group became significant when pooling partial correlations together. Thus, covariates that may affect hippocampal size such as years of education, age, gender or total intracranial volume may have an impact on verbal learning ability in healthy controls but the covariates from the different studies were too varied to derive a meaningful conclusion.

Previous investigations into the structure-function relationship have often revealed positive correlations between hippocampal volume and declarative memory performance only in the presence of pathology (e.g. Bonner-Jackson et al., 2015). However, a *meta*-analysis in healthy participants revealed a high degree of variability in the relationship between hippocampal volume and episodic memory in healthy participants where the relationship was negative in children, adolescents and young adults and almost absent in older participants (Van Petten, 2004). Thus, the absence of correlation in the current *meta*-analysis is consistent with previous findings that structure-behaviour relationships occur in the presence of pathology and could be due to the large variability in mean age of the healthy control participants (mean age range in current *meta*-analysis: 20–73) such that any effect in younger participants could be masked by the absence of effect in older participants. Furthermore, results from the *meta*-regression suggest that age only had an effect on the relationship between the right hippocampal volume and immediate recall in healthy controls.

In GHR subjects, only the left hippocampal volume was correlated with immediate recall. However, the number of studies in this analysis was very small (n = 2) and should therefore be treated with caution. This finding is consistent with a study showing that verbal learning is lateralised to the left anterior hippocampus (Persson and Soderlund, 2015). Although these participants were not symptomatic at the time of the studies, it is interesting to see that they have a similar relationship between verbal learning and left hippocampal volume to that seen in patients with schizophrenia. This suggests that some genetic factors may be influencing hippocampal volume, which is consistent with a recent study showing an association between hippocampal volume and the Polygenic Schizophrenia-related Risk Score (Harrisberger et al., 2016).

Functional imaging studies in patients with and at risk for schizophrenia consistently report changes in activation during verbal encoding and retrieval in the hippocampus and regions of the prefrontal cortex (Allen et al., 2011, Hofer et al., 2003, Kubicki et al., 2003, Ragland et al., 2001, Zierhut et al., 2010). Further studies also suggest abnormal resting state connectivity between the hippocampus and regions of the prefrontal cortex (Knochel et al., 2014, Kuhn and Gallinat, 2013, Cui et al., 2015, Zhou et al., 2008, Kraguljac et al., 2014, Duan et al., 2015, Tang et al., 2015). The same patients who had abnormal connectivity between the hippocampus and prefrontal cortex also had reduced hippocampal volume and abnormal white matter fibre integrity in the tracts connecting the hippocampus to regions of the frontal lobe (Knochel et al., 2014). Therefore, deficits in verbal memory may be related to a reduced connectivity between the hippocampus and prefrontal cortex secondary to a reduction of hippocampal volume.

Hippocampal volume reductions have been consistently reported in schizophrenia (Van Erp et al., 2016) with volume reductions spreading throughout the subfields as the illness becomes more chronic (Ho et al., 2016). Previous studies suggest that hippocampal atrophy in patients with schizophrenia may arise from an imbalance in excitatory and inhibitory transmission (Schobel et al., 2013) secondary to a reduced functioning of N-methyl-d-aspartate (NMDA) receptors and a loss of parvalbumin-containing GABAergic interneurons (Braun et al., 2007, Schobel et al., 2013, Lisman et al., 2008). GABAergic interneurons are important for synchronising the activity of glutamatergic pyramidal cells via the production of gamma oscillations (Gonzalez-Burgos and Lewis, 2012), a mechanism by which they help regulate cognitive functions such as learning and memory but may also be important for the retrieval of information (Sejnowski and Paulsen, 2006).

Whilst long-term use of antipsychotic medication has been associated with a decline in verbal learning and memory (Husa et al., 2014), more effective treatments may aim to reduce hippocampal volume loss or normalise hippocampal activity or the connectivity between the hippocampus and frontal lobe regions. Antidepressants are able to stimulate hippocampal neurogenesis and increase GABA levels (Malberg et al., 2000, Simpson et al., 2012) and may enhance long term memory (Bowie et al., 2012). Certain cholinergic agonists have shown efficacy at improving cognition and function in patients with schizophrenia (Keefe et al., 2015) whilst a partial agonist improved verbal learning and working memory (Haig et al., 2016). Other factors that can affect hippocampal volume and are common in patients with schizophrenia are the use of cannabis or the presence of chronic stress (Schlosser et al., 2012, Fusar-Poli et al., 2005, Barha et al., 2011, Demirakca et al., 2011). Therefore, therapies that help manage stress or reduce the intake of cannabis in this population may lead to beneficial improvements in verbal memory.

From the results, it is clear that covariates may influence the relationship between verbal learning and hippocampal volume differently in patients with schizophrenia and in healthy controls and the inability to properly examine the effects of these covariates suggests that the results should be treated with caution. Also, by restricting the focus of this *meta*-analysis to verbal learning and the hippocampus; it is not possible to know whether hippocampal volume abnormalities are related to other deficits in cognitive abilities or whether verbal learning relates to volume changes in other brain regions.

One of the limitations of this study is the variation in hippocampal segmentation methodologies and thus in the definition of the boundaries of the structure which in turn would affect volume measurements. Furthermore, some studies chose to divide the hippocampus into anterior and posterior regions, which is unlikely to be consistent across studies. Similarly, not all studies used absolute volume in their correlations although some studies added total intracranial volume as a covariate instead. There was also variation in the average age of the patients across the studies (age range 20–65) which itself affects regional volume and cognitive abilities (Driscoll et al., 2003) and may make the studies less comparable. However, studies examining cognitive decline over time have shown that patients with schizophrenia decline at a similar rate to healthy controls (Rannikko et al., 2015, Rajji et al., 2013).

Antipsychotic medication is also known to affect hippocampal volume (Zierhut et al., 2013, Yang et al., 2015) and hippocampal neurogenesis (Newton and Duman, 2007) which contributes to learning and memory (Deng et al., 2010, Lazarov and Hollands, 2016). However, only a proportion of patients from all studies were being treated with antipsychotic medication. Furthermore, the results from the *meta*-regressions suggest that antipsychotic medication may not be influencing the results of this *meta*-analysis.

Another limitation arises from the design of the tasks used to measure verbal learning abilities. Delayed recall is only measured after around a 20-min break; therefore such tasks do not allow for the assessment of longitudinal recall of information over months or years. Although the studies are relatively homogeneous in the tasks used, some tasks may involve slightly different cognitive demands. For example, the CVLT and HVLT have groups of words that are semantically organised which may improve recall (Poirier and Saint-Aubin, 1995) and may recruit a larger network of regions (Binder and Desai, 2011) than the RAVLT which contains unrelated words. Therefore, future research may consider the relationship between hippocampal volumes and individual tasks.

Future research should also examine the relationship between hippocampal subfields and different aspects of memory (e.g. encoding vs. retrieval) because there were insufficient studies reporting findings in hippocampal subfields to examine this in this *meta*-analysis. One could expect a higher correlation between immediate recall and Cornu Ammonis/dentate gyrus regions and between delayed recall and the subiculum (Persson and Soderlund, 2015, Zeineh et al., 2003, Eldridge et al., 2005). Finally, it would be interesting to study these effects in a sample of subjects at ultra-high risk for psychosis as this group has a higher risk of transitioning to frank psychosis compared to GHR subjects (Fusar-Poli et al., 2016).

In conclusion, patients with schizophrenia show variations in bilateral hippocampal volume that are associated with deficits in verbal learning memory. This supports a role for the hippocampus in the development of some of the cognitive symptoms observed in schizophrenia.

## Conflict of interest

None.

## Disclosure

None.
