# Supplementary material for: Verbal learning and hippocampal dysfunction in schizophrenia: A meta-analysis
Source: Neurosci Biobehav Rev. 2018 Mar;86:166–75. doi: 10.1016/j.neubiorev.2017.12.001 (PMC5818020; doi:10.1016/j.neubiorev.2017.12.001)
Supplement: Supplementary file 1 [file mmc1.docx]

Table 1. Description of the MRI segmentation protocols and regions of interest used by the studies included in the meta-analysis.

| **Study** | **Field strength** | **Segmentation method** | **Regions of interest** | **Absolute volume?** |
| --- | --- | --- | --- | --- |
| **Herold et al., 2015** | 3T | FSL’s FIRST (mesh model based tool to segment subcortical structures). | The hippocampus volume includes the dentate gyrus, CA1-4, the prosubiculum and the subiculum. | Yes (mm3) |
| **Haukvik et al., 2015** | 1.5T | FreeSurfer 5.2.0 | Hippocampal subfield volumes were estimated but only the subiculum volume was used in analyses. | Total subiculum/ICV |
| **Guo et al., 2014** | 3T | All T1 images segmented in SPM8 into GM, WM and CSF then smoothed with 8mm FWHM. Volume extracted from cluster that was significant in whole-brain voxel based morphometry. | Bilateral hippocampal gyri | Sum of voxels in significant cluster (unclear whether voxel size is 1x1x1mm) |
| **Lappin et al., 2014** | 1.5T | FreeSurfer 5.0 | Bilateral hippocampus | Yes (mm3) |
| **Francis et al., 2013** | 3T | FreeSurfer 5.0 | Bilateral hippocampi, entorhinal cortices and hippocampal subfields including CA1-3, dentate gyrus-CA4, subiculum, presubiculum and fimbria | Yes (mm3) |
| **Karnik-Henry et al., 2012** | 1.5T | HDBM-LD was used to map the hippocampal structure in each subject and then used to calculate volume. Manual definition of parahippocampal gyrus. | Bilateral hippocampus | Yes |
| **Killgore et al., 2009** | 1.5T | Manual tracing (ICC >.90) | Bilateral hippocampus and amygdala | No (proportion of ICV) |
| **Thoma et al., 2009** | 1.5T | Manual tracing on k-means segmented coronal T1-weighted images (alpha=.82). They used the mean measurement of the 2 raters. | Hippocampus volume was determined for total, right, left, anterior (anterior 9 slices), and posterior (posterior 9 slices) | Yes (mm3) |
| **Exner et al., 2008** | 1.5T | Manual tracing (using CURRY software) ICC=.91 | Bilateral hippocampus | Yes (mm3) |
| **Rametti et al., 2007** | 1.5T | Stereological analysis using ANALYZE to measure hippocampal volume. | Bilateral anterior and posterior hippocampi | mm3/ICV (units in mm3 x 100) |
| **Kuroki et al., 2006** | 1.5T | Manual tracing  (ICC .95 for the left hippocampus and .93 for the right hippocampus) | Bilateral hippocampus which includes Ammon’s horn, the dentate gyrus and the subiculum. | Yes (mL) |
| **Toulopoulou et al., 2004** | 1.5T | Manual segmentation using the MEASURE software to calculate volume. | Bilateral hippocampus | Yes |
| **Sanfilipo et al., 2002** | 1.5T | Manual tracing (ICC= .92) | Bilateral hippocampus | Absolute but corrected for ICV in analyses |
| **Seidman et al., 2002** | 1.5T | Manual segmentation (ICC 0.91 for the left hippocampus, and 0.92 for the right hippocampus) | Bilateral hippocampus | Yes (cm3) |
| **O’Driscoll et al., 2001** | 1.5T | Manual tracing (L AAH ICC = .80, R AAH ICC = .92, L posterior ICC = .95, R posterior = .75). | Amygdala-anterior hippocampus (AAH, includes anterior subiculum) and the posterior hippocampus (includes the body and tail of the hippocampus). | Yes (cm3) |
| **Gur et al., 2000** | 1.5T | Manual tracing (ICC > .90) | Bilateral hippocampi | Yes (mL) |
| **Sachdev et al., 2000** | 1.5T | Manual tracing (ICC = .98) | Bilateral hippocampus (includes the Cornu Ammonis, dentate gyrus, subiculum, alveus and fimbria). | Yes (cm3) |

ICC: Intraclass Correlation Coefficient, HDBM-LD: large deformation high-dimensional brain mapping, ICV: intracranial volume, CA: Cornu Ammonis, mL: millilitre, L: left, R: right, AAH: Amygdala-anterior hippocampus

Table 2. Summary of extracted mean left and right hippocampal volume and mean memory scores in patients with schizophrenia and their relatives, scaled as a percentage of the values and scores of healthy controls (healthy controls are 100%).

| **Study** | **Mean Left hippocampal volume (% of HC)** | **Mean Right hippocampal volume**  **(% of HC)** | **Mean Immediate memory score**  **(% of HC)** | **Mean Delayed memory score**  **(% of HC)** |
| --- | --- | --- | --- | --- |
| Exner et al., 2008 Males | 79.0 | 77.7 | 73.5 | 61.3 |
| Exner et al., 2008 Females | 104.1 | 100 | 74.3 | 76.7 |
| Francis et al., 2013 | 94.3 | 94.0 | 89.3 | 96.2 |
| Haukvik et al., 2015 | 94.9 | 94.1 | 84.5 | 84.3 |
| Herold et al., 2015  Old | 86.2 | 91.5 | 48.7 | 38.7 |
| Herold et al., 2015  Young | 79.1 | 102.0 | 70.3 | 63.4 |
| Driscoll et al., 2001 Anterior | 91.7 | 91.6 | 88.5 | 81.0 |
| Driscoll et al., 2001  Posterior | 103.6 | 96.8 | 88.5 | 81.0 |
| Rametti et al., 2007 | 99.1 | 97.2 | 86.3 | 81.5 |
| Seidman et al., 2002  Simplex | 95.2 | 103.3 | 97.9 | 99.6 |
| Seidman et al., 2002  Multiplex | 90.6 | 100.3 | 77.8 | 77.6 |
| Sachdev et al., 2000 | 91.1 | 93.1 | - | 49.0 |
