# Supplementary material for: Verbal learning and hippocampal dysfunction in schizophrenia: A meta-analysis
Source: Neurosci Biobehav Rev. 2018 Mar;86:166–75. doi: 10.1016/j.neubiorev.2017.12.001 (PMC5818020; doi:10.1016/j.neubiorev.2017.12.001)
Supplement: Supplementary file 2 [file mmc2.docx]

Figure 1. Forest plot of the association between left hippocampal volume and immediate recall in patients with schizophrenia. Each data marker represents a study, its reported correlation coefficient and the 95% confidence interval. The size of the data marker is proportional to the total number of individuals in that study. Asterisks denote p < 0.05.

Figure 2. Forest plot of the association between left hippocampal volume and delayed recall in patients with schizophrenia. Each data marker represents a study, its reported correlation coefficient and the 95% confidence interval. The size of the data marker is proportional to the total number of individuals in that study. Asterisks denote p < 0.05 and dots represent trend level significance.

Figure 3. Forest plot of the association between right hippocampal volume and immediate recall in patients with schizophrenia. Each data marker represents a study, its reported correlation coefficient and the 95% confidence interval. The size of the data marker is proportional to the total number of individuals in that study. Asterisks denote p < 0.05 and dots represent trend level significance.

Figure 4. Forest plot of the association between right hippocampal volume and delayed recall in patients with schizophrenia. Each data marker represents a study, its reported correlation coefficient and the 95% confidence interval. The size of the data marker is proportional to the total number of individuals in that study. Asterisks denote p < 0.05.

Figure 5. Forest plot of the association between left hippocampal volume and immediate recall in healthy controls. Each data marker represents a study, its reported correlation coefficient and the 95% confidence interval. The size of the data marker is proportional to the total number of individuals in that study. Asterisk denote p < 0.05 and dots represent trend level significance. Studies with NSUEs are coloured in grey and the light grey shadow area represents the interval containing 95% of the imputed correlation coefficients and the dark grey circle within the shaded area represents the mean.

Figure 6. Forest plot of the association between left hippocampal volume and delayed recall in healthy controls. Each data marker represents a study, its reported correlation coefficient and the 95% confidence interval. The size of the data marker is proportional to the total number of individuals in that study. Dots on the right hand side represent trend level significance. Studies with NSUEs are coloured in grey and the light grey shadow area represents the interval containing 95% of the imputed correlation coefficients and the dark grey circle within the shaded area represents the mean.

Figure 7. Forest plot of the association between right hippocampal volume and immediate recall in healthy controls. Each data marker represents a study, its reported correlation coefficient and the 95% confidence interval. The size of the data marker is proportional to the total number of individuals in that study. Dots on the right-hand side represent trend level significance. Studies with NSUEs are coloured in grey and the light grey shadow area represents the interval containing 95% of the imputed correlation coefficients and the dark grey circle within the shaded area represents the mean.

Figure 8. Forest plot of the association between right hippocampal volume and delayed recall in healthy controls. Each data marker represents a study, its reported correlation coefficient and the 95% confidence interval. The size of the data marker is proportional to the total number of individuals in that study. Asterisk denote p < 0.05 and dots represent trend level significance. Studies with NSUEs are coloured in grey and the light grey shadow area represents the interval containing 95% of the imputed correlation coefficients and the dark grey circle within the shaded area represents the mean.
